# Tea drinking habits and osteoporotic hip/femur fractures: A case-control study

**DOI:** 10.12669/pjms.322.9092

**Published:** 2016

**Authors:** Chenshu Huang, Rongrui Tang

**Affiliations:** 1 Chenshu Huang, PhD, MD. Department of Radiation Medicine, Shapingba Hospital, Shapingba District, Chongqing, China; 2 Rongrui Tang, Professor, Department of Radiation Medicine, Shapingba Hospital, Shapingba District, Chongqing, China

**Keywords:** Tea, Hip, Fracture, Case-control study, Osteoporosis

## Abstract

**Objective:**

To explore the relationship between tea drinking habits and osteoporotic hip/femur fractures.

**Methods:**

Paired case-control method was used for face-to-face interviews from January 2010 to June 2014. Patients (n=435) with newly osteoporotic hip/femur fracture and 435 controls with the same gender and age (±3) were given questionnaire survey. The survey content included general situation, detailed tea drinking and other diet condition, health-related behavior and family history of fractures, etc.

**Results:**

Single factor logistic analysis showed that the habit of drinking tea can significantly reduce the risk of hip/femur fracture. Cumulative year of tea drinking, the cumulative amount of tea and tea concentration (low dose group) have the maximum protection for fracture, while the high dose group is weaker in protection (trend test, P<0.05). After adjustment for age, energy, BMI, education degree, parents’ history of fracture, second hand smoke exposure, calcium supplements, and equivalent energy consumption of physical activity, etc, the above association still showed significant linear trend, but the associated strength was slightly reduced. But stratified analysis found that the effect of tea drinking was only statistically significant in men. And there were no statistically significant differences of people with different education degree.

**Conclusions:**

Regular tea drinking can reduce the risk of osteoporotic hip/femur fractures in middle-aged and elderly men.

## INTRODUCTION

Osteoporosis (OP) is a kind of systemic disease that is prone to damage of bone tissue micro-structure, reduced bone strength and increased osteopsathyrosis.^1^ It may cause osteoporotic fracture, especially hip/femur fracture and has become a serious worldwide public health problem with higher incidence, mortality and disability.^2^ The protective role of tea and its extracts on the bone is related to the effects that have been put forward.^3^ Compared with non-tea drinkers, tea drinkers have better bone mineral density (BMD) at each part,^4^ but the relationship between tea drinking and osteoporotic hip/femur fracture is not cleay. This study aimed at analysis on relationship between tea drinking and osteoporotic fracture.

## METHODS

### Research objects

Patients (n=435) with newly osteoporotic hip/femur fracture (violent or high-energy fractures are excluded) were enrolled as cases from our hospital and several affiliated hospitals between January 2010 and June 2014 based on the X-ray diagnosis. New case was defined as the patient admitted within one week after femoral neck fracture and intertrochanteric fractures. With case group as the reference standard, 435 community residents or cases with non-orthopedic related disease were recruited as the controls from the above hospital physical examination center and the surrounding communities over the same period. The patients were aged between 58 and 82 years of age, and did not have history of fracture. The cases and controls were paired at 1:1 in accordance with the same gender and age (±3), which were excluded for serious diseases (such as pathological fracture, high-energy fractures, stroke, cancer and liver cirrhosis) and mental or cognitive disorders, unable to walk normally, blind at bilateral eyes, premenopausal hysterectomy or ovariectomy, and diabetes diet therapy. This study was approved by ethical committee of our hospital, and the research subjects signed informed consent prior to the survey.

### Survey methods and quality control

Questionnaire of unified design was adopted. And the same method was adopted in both the groups for investigation. Survey items included general demographic information. The relevant variables of this research were defined as: tea drinking referred to drink at least onecup of tea every week for three months or more; average number of tea drinking weekly was defined as the number of tea drinking every week based on the tea drinking habit of the respondents. The participators with regular tea drinking history were explored in detail for their accumulate time of tea drinking, the average number per week, the average amount per year (low dose: less than 5 kilogram; moderate dose: more than 5 kilogram and less than 10 kilogram; high dose: more than 10 kilogram), tea concentration, and total amount of tea drinking which is the result of average amount per year multiplying with total time.

### Statistical analysis

Conditional logistic regression was used for single factor and multiple factors analysis to discuss the relationship between tea drinking and osteoporotic fractures. Multiple factors analysis and Multi-factor model were used to test the interaction effects on hip fracture risk. SPSS 16.0 was used for statistical analysis. Bilateral inspection was used for all the statistical tests and P<0.05 refers to statistically significant difference.

## RESULTS

### General condition

There were a total of 435 pairs in the case group and control group. Compared with the case group, control group had higher BMI, education degree and average daily intake of energy, and higher proportion of calcium tablet intake and physical activity, but the proportions of family history of fractures and of the second hand smoke exposure were low. Table-I

**Table-I:** General Condition

Variable	The Controls Group (n=435)	The Cases Group (n=435)
*Gender*		
Male / Female	322/113	322/113
Age (Mean±sd)	71.31±6.96	71.93±7.23
Body mass index (Mean±sd, kg/m2)	22.52±3.74	21.26±3.54
*Tea drinking habits*		
Tea drinking habits Yes	191 (44.0%)	149 (34.3%)
No	244 (56.1%)	286 (65.7%)
Cumulative year of tea drinking (Mean±sd)	11.28±14.92	9.15±13.83
Average time per week (Mean±sd)	2.20±2.89	1.67±2.67
Average amount of tea drinking per year (Mean±sd, kg)	0.67±1.35	0.50±0.88
Cumulative amount of tea (Mean±sd, kg)	12.82±28.34	15.64±38.36
*Tea concentration*		
Tea concentration No	233 (56.0%)	272 (65.4%)
Low dose	75 (18.0%)	50 (12.0%)
Medium and High dose	108 (26.0%)	94 (22.6%)

***Note:*** Data out of the parentheses is the number of cases, while data in the parentheses is the proportion (%)

### Relationship between tea drinking and osteoporotic hip/femur fractures

Single factor logistic analysis showed that tea significantly reduce the risk of hip fracture. Longer tea drinking year, more times of tea drinking every week, larger amount of tea drinking every year, larger total amount of tea drinking, or lower tea concentration, might lead to lower risk of fractures. Compared with non-tea drinker, OR value of tea drinking (different time, frequency, tea dose) was 0.54 ~ 0.84. After adjustment for age, energy, BMI, education degree, parents’ history of fracture, second hand smoke exposure, calcium supplements, and equivalent energy consumption of physical activity, etc, the associated strength was slightly reduced. Table-II

**Table-II:** Relationship between Tea drinking and osteoporotic Hip/femur Fractures.

Variable	Number of Cases/controls	Single factor analysis		Multiple factors analysis	

		OR (95%CI)	p-Value	OR (95%CI)	p-Value
Tea drinking habits					
Tea drinking habits No	286/244	1.00	0.001	1.00	0.021
Yes	149/191	0.66(0.52~0.84)		0.72(0.54 ~ 0.95)	
Cumulative year of tea drinking			0.002		0.034
No	286/244	1.00		1.00	
≤20y	75/95	0.68(0.48 ~ 0.91)		0.71(0.50 ~ 0.96)	
>20y	74/96	0.69(0.48 ~0.90)		0.73(0.52 ~ 1.08)	
The average weekly frequency			0.001		0.034
0	286/244	1.00		1.00	
1-6	74/97	0.66(0.51 ~ 0.91)		0.72(0.45 ~ 0.98)	
≥7	75/94	0.65(0.46 ~ 0.91)		0.72(0.49 ~ 1.08)	
The average weekly frequency			0.002		0.031
No	286/244	1.00		1.00	
≤median	98/113	0.75(0.56 ~ 0.98)		0.84(0.61 ~ 1.16)	
>median	51/78	0.54(0.38 ~ 0.77)		0.54(0.36 ~ 0.83)	
Cumulative amount of tea			0.004		0.062
No	286/244	1.00		1.00	
≤median	78/100	0.65(0.48 ~ 0.89)		0.71(0.48 ~ 0.96)	
>median	71/91	0.66(0.48 ~ 0.92)		0.75(0.55 ~ 1.09)	
Tea concentration			0.005		0.043
No	286/244	1.00		1.00	
Low dose	52/77	0.56(0.40 ~ 0.78)		0.62(0.42 ~ 0.95)	
Medium and High dose	97/114	0.72(0.54 ~ 0.95)		0.75(0.55 ~ 1.08)	

***Note:*** Conditional logistic regression is adopted for data analysis. Correction factors of multiple factors analysis.

### Interactive analysis

Interactive analysis of gender and tea drinking showed that the differences of the associated intensity were not statistically significant (p=0.343 ~ 0.953). But stratified analysis found that the effect of middle dose group is superior in men to women (as shown in Table-III). Tea drinking year of men ≤ 20 years (OR=0.21, 95% CI:0.07~0.67), tea drinking frequency of l~6 times per week(OR=0.32, 95%CI:0.12~0.87), total amount of tea drinking less than the median(OR=0.31, 95%CI:0.12~0.77). There were no statistical correlations in women between tea drinking and fracture. There was no significant association of education and tea drinking for hip fracture (p>0.05).

**Table-III:** Interactive Analysis of Gender and Tea Drinking.

Variable	Female (n= 322)		Male (n=113)		Interactive analysis

	OR(95%CI)	p-Value	OR(95%CI)	p-Value	p-Value
Tea drinking habits	0.621		0.110	0.417	
No	1.00		1.00		
Yes	0.95(0.69~1.37)		0.52(0.23~1.17)		
Cumulative year of tea drinking		0.693		0.435	0.946
No	1.00		1.00		
≤20y	1.06(0.72~1.60)		0.21(0.07~0.67)		
>20y	0.87(0.52~1.47)		0.69(0.28~1.67)		
Average time per week		0.602		0.346	0.575
0	1.00		1.00		
1-6	1.26(0.72~1.77)		0.32(0.12~0.87)		
≥7	0.85(0.62~1.47)		0.82(0.32~2.57)		
Average time per week		0.502		0.053	0.343
No	1.00		1.00		
≤median	1.26(0.72~1.77)		0.62(0.72~1.46)		
>median	0.85(0.62~1.41)		0.42(0.72~1.17)		
Cumulative amount of tea		0.673		0.463	0.953
No	1.00		1.00		
≤median(13.5kg)	1.16(0.72~1.77)		0.31(0.12~0.77)		
>median(13.5kg)	0.85(0.62~1.37)		0.72(0.32~1.82)		
Tea categories					0.423
No	1.00		1.00		
Green tea	0.96(0.72~1.82)		0.51(0.31~0.90)		
Black tea	0.93(0.60~1.44)		0.45(0.22~0.93)		
Oolong tea	1.05(0.55~2.07)		0.40(0.14~1.07)		
Tea concentration		0.775		0.243	0.467
No	1.00		1.00		
Low dose	0.91(0.55~1.42)		0.32(0.12~1.07)		
Medium and High dose	0.96(0.67~1.77)		0.62(0.25~1.62)		

***Note:*** paired t test (continuous variables) and paired χ^2^ test (classification variables) are adopted for comparison of the two groups.

## DISCUSSION

Effects of different tea components on bone metabolism have been studied from tissue morphometry, bone cytology, epidemiological surveys and clinical experiments in recent years.^2^, ^5^-^8^ The main components of tea are alkaloids, tea polyphenols, mineral elements, etc^3^, which contain a variety of active ingredients that can affect bone metabolism, such as tea polyphenols, caffeine, fluoride, etc. They have obvious effects on the activity of osteogenesis and osteoclasts and bone transformation and absorption via different channels.^4^

This study proves that habit of tea drinking is a protective factor for osteoporotic fractures. Positive effect of tea drinking habit on bone density may be related to antioxidant effect of tea polyphenols, mild activation of estrogen, and effects of fluoride.^4^ Previous studies usually use BMD and bone mineral content as the research objects, and mainly focus on the relationship between tea drinking with osteoporosis.^5^, ^6^ Devine’s study^1^ confirmed that there was a positive correlation between tea drinking and bone mass maintenance, and the bone density of the tea drinker was often higher than that of the non-tea drinkers. Hegarty’s study^4^ showed that bone density of the black tea drinkers was higher than the non-tea drinkers by about 2.8% ~ 5%. There were linear correlations between tea dose with bone density at lumbar vertebrae, femoral trochanter and Ward’s triangle region of postmenopausal women. Wu^8^ confirmed that there was a positive correlation between tea drinking years of the adult women with their bone density. For women drinking tea for more than 10 years, bone density of their whole body, lumbar vertebrae, and femur was all increased; while for women drinking tea for 5~10 years, only the bone density of lumbar vertebrae was increased. In animal experiment, composition of tea was also closely related to bone metabolism index.^6^, ^7^

This study found that the high concentration group was weaker in protection. The reason for this may be related to the adverse effects of caffeine in tea on bone health.^9^, ^10^ Because caffeine accounts for 2% ~ 4% of the dry weight of the tea, drinking more tea will intake more caffeine.^11^, ^12^ Caffeine becomes agonist of adenylate cyclase (route C) through inhibition on the activity of phosphodiesterase, which affects on the bone. In addition, caffeine can increase urinary calcium excretion and reduce the intestinal absorption of calcium, leading to negative calcium balance thus promoting increased bone absorption. Heaney’s research suggested that long-term high doses of caffeine intake can (> 300 mg/d) directly produce negative effect on bone mineral density, and increase the fracture risk at lumbar vertebrae, femoral neck and other parts.^13^ Xiang found^11^ that postmenopausal women with drinking caffeinated drinks daily may increase bone loss. Tannic acid also reduces the absorption of calcium, thus affecting BMD.^12^

This study also found that the protective effects of tea drinking on fracture are only statistically significant in the male. Physiological characteristics and the endocrine metabolism differences between genders may also be the reason. Sasazuki^14^ have reported that in Japanese people it was found that green tea played a protective role for male coronary atherosclerosis, but this relationship was not found among women. In addition, protective effects of drinking tea on female fracture were also not statistically significant in the United States^3^, Turkey^2^, and Sweden.^15^ In this paper, as for the males, tea drinking amount and time were both higher than the females, and the different exposures can also another reason for effect differences.

## CONCLUSION

This retrospective study can therefore only provide preliminary data of association of tea drinking habits and osteoporotic hip/femur fractures. However, our findings serve as a valuable attempt for the better understanding of tea drinking habits and osteoporotic hip/femur fractures to explore the prevention and cure strategy of osteoporosis fracture.

## References

[1] Devine A, Hodgson JM, Dick IM, Prince RL (2007). Tea drinkings associated with benefits on bone density in older women. Am J Clin Nutr.

[2] Hamdi KI, Aydin SI, Gemalmaz A (2007). Habitual tea drinking and bone mineral density in post menopausal Turkish women: investigation of prevalence of postmenopausal osteoporosis in Turkey (IPPOT Study). Int J Vitam Nutr Res.

[3] Chen Z, Pettinger MB, Ritenbaugh C (2003). Habitual tea consumption and risk of osteoporosis: a prospective study in the women health initiative observational Cohort. Am J Epidemiol.

[4] Hegarty VIM, May HM, Khaw KT (2000). Tea drinking and bone mineral density in older women. Am J Clin Nutr.

[5] Wu CH, Yang YC, Yao WJ, Lu FH, Wu JS, Chang CJ (2002). Epidemiological evidence of increased bone mineral density in habitual tea drinkers. Arch Intern Med.

[6] Shen CL, Yeh JK, Cao JJ, Chyu MC, Wang JS (2011). Green tea and bone health: Evidence from laboratory studies. Pharmacol Res.

[7] Ko CH, Lau KM, Choy WY, Leung PC (2009). Effects of tea catechins, epigallocatechin, gallocatechin and gallocatechin gallate, on bone metabolism. J Agric Food Chem.

[8] Wu CH, Yang YC, Yao WJ, Lu FH, Wu JS, Chang CJ (2002). Epidemiological evidence of increased bone mineral density in habitual tea drinkers. Arch Intem Med.

[9] Harris SS, Dawson Hughes B (1994). Caffeine and bone loss in healthy postmenopausal women. Am J Clin Nutr.

[10] Zhai FY, Yang XG (2006). Report 2 for the survey of nutrition and health in Chinese residents: consumption of diet and nutrients in 2002.

[11] Xiang LW (2009). The processing effect on caffeine content in oolong tea. Amino Acids Biotic Res.

[12] Namkung W, Thiagarajah JR, Phuan PW, Verkman AS (2010). Inhibition of Ca2. activated Cl-channels by gallotannins as a possible molecular basis for health benefits of red wine and green tea. Fas Eb J.

[13] Heaney RP (2002). Effects of caffeine on bone and the calcium economy. Food Chem Toxicol.

[14] Sasazuki S, Kodama H, Yoshimasu K (2000). Relation between green tea consumption and the severity of coronary at herosclerosis among Japanese men and women. Ann Epidemiol.

[15] Hallstrom H, Wolk A, Glynn A, Michaelsson K (2006). Coffee, tea and caffeine consumption in relation to osteoporotic fracture risk in a cohort of Swedish women. Osteoporos Int.

